# Sodium Selenate, Potassium Hydroxy-Selenide, Acetylselenide and Their Effect on Antioxidant Metabolism and Plant Nutrition and Yield in Sorghum Genotypes

**DOI:** 10.3390/foods12102034

**Published:** 2023-05-17

**Authors:** Patriciani Estela Cipriano, Rodrigo Fonseca da Silva, Cynthia de Oliveira, Alexandre Boari de Lima, Fabio Aurélio Dias Martins, Gizele Celante, Alcindo Aparecido dos Santos, Marcos Vinicio Lopes Rodrigues Archilha, Marcos Felipe Pinatto Botelho, Valdemar Faquin, Luiz Roberto Guimarães Guilherme

**Affiliations:** 1 Department of Soil Science, Federal University of Lavras, Lavras 37200-900, MG, Brazil; patricianiestela@gmail.com (P.E.C.); rodrigo11.07@hotmail.com (R.F.d.S.); cynthiaoliveiraufla@gmail.com (C.d.O.); alexandre.boari@ufla.br (A.B.d.L.); vafaquin@ufla.br (V.F.); 2 Minas Gerais Agricultural Research Agency, Experimental Field of Maria da Fé, Maria da Fé 37517-000, MG, Brazil; 3 Minas Gerais Agricultural Research Agency, Experimental Field of Lavras, Lavras 37200-900, MG, Brazil; fabio.aurelio@epamig.br; 4 Institute of Chemistry, University of São Paulo, Butantã 05508-000, SP, Brazil; celante.g@gmail.com (G.C.); alcindo@iq.usp.br (A.A.d.S.); archilha@selenolife.com.br (M.V.L.R.A.); marcospinatto@gmail.com (M.F.P.B.); 5 SelenoLife Selênio P/Vida Ltda, Butantã 05508-000, SP, Brazil

**Keywords:** selenate, selenide, food security, food composition

## Abstract

Agronomic biofortification with selenium (Se) effectively reduces hidden hunger and increases the nutritional intake of Se in people and animals. Because sorghum is a staple diet for millions of people and is used in animal feed, it becomes a crop with biofortification potential. Consequently, this study aimed to compare organoselenium compounds with selenate, which is effective in numerous crops, and to assess grain yield, the effect in the antioxidant system, and macronutrient/micronutrient contents of different sorghum genotypes treated with Se, via foliar spray. The trials used a 4 × 8 factorial design, with four Se sources (control—without Se supply, sodium selenate, potassium hydroxy-selenide, acetylselenide) and eight genotypes (BM737, BRS310, Enforcer, K200, Nugrain320, Nugrain420, Nugrain430, and SHS410). The Se rate used was 0.125 mg plant^−1^. All genotypes reacted effectively to foliar fertilization with Se through sodium selenate. In this experiment, potassium hydroxy-selenide and acetylselenide showed low Se levels and lower Se uptake and absorption efficiency than selenate. Selenium fertilization increased grain yield and altered lipid peroxidation by malondialdehyde content, hydrogen peroxide content, catalase activity, ascorbate peroxidase, superoxide dismutase, and macronutrients and micronutrients content of the studied genotypes. In sum, biofortification with selenium led to an overall yield increase of sorghum plants and supplementation with selenium through sodium selenate was more efficient than organoselenium compounds, yet acetylselenide had a positive effect on the antioxidant system. Sorghum can be effectively biofortified through the foliar application of sodium selenate; however, studying the interaction between organic and inorganic Se compounds in plants is necessary.

## 1. Introduction

Over 500 million people rely on sorghum (*Sorghum bicolor* L. Moench) as a main meal [1]. Sorghum production in Brazil was 2.7 million tons on 818.3 thousand hectares in 2019, whereas global production was around 57.9 million tons on 40 million hectares [2]. Sorghum possesses drought resilience, high productivity, a minimal demand for mineral nutrition, and low production costs, allowing it to be grown in a variety of conditions across the world [3]. According to Lopes et al. [4], sorghum may be introduced to the diet by ingesting sorghum flour, which benefits individuals by enhancing antioxidant and modulating status. Research shows that whole sorghum is a reasonable basis for carbohydrates, fiber, bioactive components (phenolic acids and anthocyanins), starch, and minerals [5].

The mineral content varies depending on the nutritional status of the plants. Nutrients and Se can have synergistic or antagonistic associations, enhancing plant nutrition and alleviating abiotic stressors such as drought, high temperatures, salt, and heavy metals [6]. Selenoproteins function as antioxidants in plant metabolism via the glutathione peroxidase (GSH) pathway, increasing enzymatic (SOD, CAT, and APX) and non-enzymatic (ascorbic acid, flavonoids, and tocopherols) compounds involved in the reactive oxygen species (ROS) elimination system and cell detoxification [7]. Different plant species have variable physiological responses to Se. Several plant species are resistant to Se and accumulate it in high amounts when grown on seleniferous soils. However, most plants are sensitive to Se and accumulate it in low amounts [8].

Selenium content in the soil is inconsistent worldwide, with soils classified as poor in Se to seleniferous soils [9]. In Brazil, a similar scenario occurs due to its broad territorial extension. Carvalho et al. [10] found Se contractions ranging from 22 to 72 µg kg^−1^ in soils of the Brazilian Cerrado. Moreover, Reis et al. [11] found Se content ranging from 0.002 to 0.65 µg kg^−1^. Soil with low Se contents causes a low content of this element in plants. Hence, soils with low Se contents yield low Se contents in plants, so Se deficiency was mainly observed in these areas and occurred in people worldwide [12]. Selenium is a micronutrient required for animal and human nutrition [8]. It is biological actions that give favorable consequences when at applicable rates.

The biological functions of Se were carried out through 25 known selenoprotein genes that encode various parts [13]; these genes are related to thyroid hormone synthesis, antioxidant defense system, and immune function [14] in animals and humans. Recently, Moghaddam et al. [15] correlated that people with adequate levels of Se had lower mortality risk if detected by SARS-CoV-2.

Dietary Se intake can be complemented by the biofortification of foods, including fertilization and genetic approaches [16]. Depending on the Se form used, selenium can be transported from the root to the shoot. Compared with selenite or organoselenium compounds, like SeMet, selenate was significantly easier to transfer [8]. To boost the Se content of farmed grains or by the foliar application of selenite or selenate, foliar fertilizers containing selenite or selenate have been used in countries including Finland, the United Kingdom, Malawi, and China [17,18,19,20]. The primary source of selenium used is sodium selenate. However, new selenium compounds have been synthesized due to their antioxidant action and the lower cost of preparing them. These compounds are potentially used in a biofortification program, but there are few studies on the fertilization of organoselenium compounds, mainly with foliar application. Consequently, this study aimed to compare organoselenium compounds with selenate, which is effective in numerous cultures, and to assess the grain yield, antioxidant system, and macronutrient/micronutrient content of different sorghum genotypes treated with foliar Se.

## 2. Materials and Methods

### 2.1. Synthesis of Selenium-Containing Compounds

Potassium hydroxy-selenide was prepared according to patent WO 2015/155453A2; Acetylselenide was prepared according to the procedure previously reported by Pinatto-Botelho et al. [21].

### 2.2. Experimental Design and Greenhouse Conditions

From February to August 2019, the research was performed in a greenhouse at the Federal University of Lavras’ Department of Soil Science. In the Soil Taxonomy [22], the soil employed in the pots matched to a dystrophic Red-Yellow Latosol [23] with a sandy, clayey loam texture and to the Typic Haplustox (loam). This study’s genotypes are commercial genotypes grown in Brazil (Table S1—Supplementary Material). Two studies were conducted at the same time and in the same way. Details on the experiment’s fertilization, management, and greenhouse conditions can be found in Cipriano et al. [24].

Eight genotypes (BM737, BRS310, Enforcer, K200, Nugrain320, Nugrain420, Nugrain430, and SHS410) were utilized with four Se sources (control—without Se supply, sodium selenate, potassium hydroxy-selenide, and acetylselenide), each with four repetitions, for a total of 128 experimental plots in a completely randomized design.

To prepare the rate of 0.125 mg plant^−1^ of Se, the sources were diluted in a 0.5% surfactant solution (Assist^®^), and two applications were made. When the plants were in the blooming stage, the first foliar treatment of Se was applied, and when the plants were in the grain-filling stage, the second foliar application of Se was applied. Just deionized water containing the surfactant was used in the control treatment. The foliar sprays were performed using a manual sprayer and preliminary compression.

### 2.3. Biochemical Analysis

On the sixth day following the second Se application, sampling was done. With rapid conditioning in liquid nitrogen and storage at −80 °C for biochemical analysis, the V2 leaves were harvested [24]. The measurement of hydrogen peroxide (H_2_O_2_) followed Velikova’s instructions [25]. The method proposed by Buege and Aust [26] was used to calculate lipid peroxidation (MDA). Superoxide dismutase (SOD, EC: 1.15.1.1), catalase (CAT, EC: 1.11.1.6), and ascorbate peroxidase (APX, EC: 1.11.1.11) activities were quantified as described by Giannopolitis and Ries [27], Havir and Mchale [28], and Nakano and Asada [29], respectively. The proteins were determined as described by Bradford [30].

### 2.4. Grain Yield

The harvested sorghum grains were weighed to calculate grain yield. The grain yield was calculated by collecting and dividing the grain produced by plants per pot by the number of plants. Grain moisture was assessed using the Seed Analysis Rule [31], and grain yield was converted to dry weight using a 13% moisture adjustment [24].

### 2.5. Sample Digestion Procedure and Nutrients Determination

After dividing the plants into grains and shoots, they were placed in a cross-airflow at around 60 °C until they attained constant weight (after about 72 h). The plants were dried and powdered. After which 0.5 g of each sample was collected for digestion using the 3051A technique specified by the USEPA [32]. The sample digestion procedure and the macronutrients, micronutrients, and Se determination was done as described by Cipriano et al. [24].

The extracts were digested and then chilled to room temperature. The extracts were then transferred to flasks and kept at 5 °C until analysis by adding 5 mL of deionized water to the total volume of the extract. By using optical emission spectrometry with inductively coupled plasma (ICP-OES), brand Spectro, and model Blue (Germany), with correction background, the contents of S, P, K, Ca, Mg, Fe, Cu, Zn, and Mn were determined with the operating parameters and the sample introduction system as specified by the manufacturer. Cipriano et al. [24] detailed the parameters, and the spectral line for each determining element is presented in Table S3—Supplemental Material.

Each batch contained a blank sample and a sample of standard reference material (Peach Leaves SRM1547) for plant material to ensure quality. By examining the standard reference material, the analytical procedure’s correctness was confirmed, and the findings showed that it was accurate to within 72% of certified values.

Kjeldahl distillation and sulfuric digestion were used to calculate the total N content [33]. Peach Leaves standard reference material (SRM1547) had a mean N recovery of 116.3% (1.93 s.e.m.).

#### Selenium (Se)

By the Selenium determination, the samples were analyzed by a Perkin-Elmer AAnalyst 800 Graphite Furnace Atomic Absorption Spectrometry—GFAAS with Zeeman background correction and EDL lamp for Se [24]. Each digestion batch contained a sample of the industry standard reference material (White Clover—BCR 402, Institute for Reference Materials and Measurements, Geel, Belgium) for quality control and a used blank sample to determine the detection limits and quantification. The average Se recovery in this standard reference material was 92.9% (*n* = 10, [Se] = 6.57 mg kg^−1^).

The limits of detection (LOD) and quantitation (LOQ) were determined using ten blank extracts in the same manner as the samples. LOD and LOQ were determined by multiplying three and ten times the standard deviation of 10 blank extracts, respectively [34]. In the research materials, the LOD was 52 g kg^−1^ of extract Se, and the LOQ was 173 g kg^−1^ of extract Se. Undiscovered values (control treatment—without Se foliar) were replaced with half the LOD (½LOD) [35].

### 2.6. Efficiency Nutritional of Se

Mathematical representations of nutritional efficiency principles offered by different scholars were used to investigate the efficiency of Se utilization by sorghum plants. Since production was quantified in g plant^−1^ [36,37], the absorption efficiency of Se rates applied (SeAE) was given in % [37]. The equations are explained further below.

(1) SeU=Se content in grain×grain yield

(2) SeAE={(SeUtreated-SeUcontrol)×100}/Se supply

### 2.7. Statistical Analysis

Initially, the normality of the data was evaluated using the Shapiro–Wilk test (*p* ≥ 0.05), homoscedasticity was assessed using the Barlet test (*p ≥* 0.05), and residue independence was checked using the Durbin-Watson test (*p ≥* 0.05). Next, a variance analysis was performed, and means were compared using the Scott-Knott test (*p ≥* 0.05). The distribution of data was visualized using box plots. To account for differences in the units of measurement, the data were normalized according to Cao et al. [38] before being agglomerated.

Principal component analysis (PCA) was used to report the variance in the genotypes for each selenium source. This approach allowed for identifying the factors that most strongly discriminated between structural features in each treatment. As a result, two additional orthogonal latent variables were defined, which helped to represent the initial set of variables in two-dimensional figures [39]. All statistical analyses and visualizations were performed using the R software [40].

## 3. Results

Except for Mn in the shoot, the sorghum plants responded based on the interaction between genotypes and Se sources (*p* ≤ 0.05) for the variables tested in antioxidant metabolism, grain yield, and macro and micronutrient content in the grain and the shoot. The grain weight and Mn content of the shoot differed considerably (*p* ≤ 0.05) across genotypes (Table S4—Supplementary Material).

### 3.1. Antioxidant Metabolism

**Figure 1:**
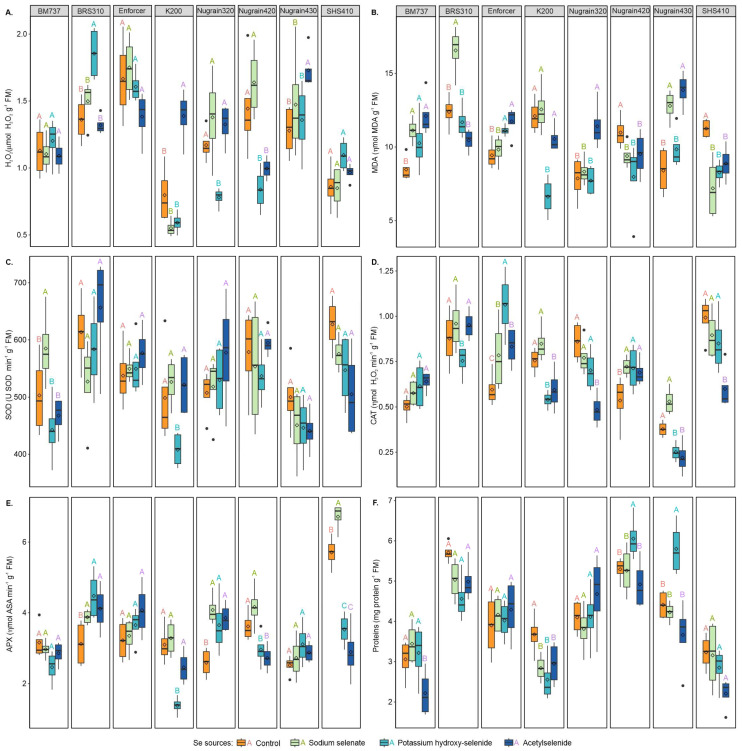
Hydrogen peroxide—H_2_O_2_ (**A**), lipid peroxidation by the malondialdehyde content—MDA (**B**), catalase—CAT (**C**), ascorbate peroxidase—APX (**D**), superoxide dismutase—SOD (**E**), and proteins in extract enzymatic (**F**). Se sources with the same genotype are compared using uppercase letters. Using the Scott-Knott test, different letters indicate significant differences between treatments at a probability threshold of 5% (*p* < 0.05).

The results presented in Figure 1A reveal that treating K200 with sodium selenate resulted in the lowest H_2_O_2_ level. Interestingly, using K200 without Se foliar supply and treatments with potassium hydroxy-selenide and sodium selenate also resulted in significantly lower H_2_O_2_ content (42.44%, 57.65%, and 60.43%, respectively) compared with the acetylselenide treatment. BRS310 exhibited also significant reductions in H_2_O_2_ content (26.48%, 18.91%, and 28.64%), when comparing the source potassium hydroxyl-selenide with the treatments without Se foliar supply (control), sodium selenate, and acetylselenide, respectively.

Compared with sodium selenate, Nugrain420 demonstrated lower H_2_O_2_ levels (48.78% and 39.02%) for the sources potassium hydroxy-selenide and acetylselenide. Moreover, for Nugrain430, the treatment without Se foliar supply, sodium selenate, or potassium hydroxy-selenide showed a lower H_2_O_2_ content than with acetylselenide, with reductions of 28.01%, 15.02%, and 21.38%, respectively. When compared with the treatments without Se foliar supply, sodium selenate, and acetylselenide, Nugrain320 treated with potassium hydroxy-selenide exhibited significant reductions in H_2_O_2_ content, with decreases of 33.33%, 43.47%, and 41.35%, respectively.

The results depicted in Figure 1B demonstrate that selenium effectively reduced lipid peroxidation, as measured by MDA levels, in SHS410 and Nugrain420. Specifically, for SHS410, treatments with sodium selenate, potassium hydroxy-selenide, and acetylselenide led to significant reductions in MDA levels compared with the treatment without Se foliar supply, with decreases of 36.06%, 26.47%, and 21.13%, respectively. Furthermore, compared with the Nugrain420 treatment without Se foliar supply, sodium selenate, potassium hydroxy-selenide, and acetylselenide treatments decreased MDA content by 14.56%, 27.48%, and 13.37%, respectively. In the case of BRS310, the application of sodium selenate led to even more significant increments in MDA content, with an increase of 25.18%, 29.41%, and 36.83% observed in the treatments without Se foliar supply, potassium hydroxy-selenide, and acetylselenide, respectively.

Finally, for K200, using potassium hydroxy-selenide resulted in significant reductions in MDA content (45.22%, 47.21%, and 37.16%) compared with the treatment without Se foliar supply, sodium selenate and acetylselenide, respectively. The MDA content of Nugrain320 was significantly lower by 31.02%, 26.99%, and 32.34% without Se foliar supply, sodium selenate, and potassium hydroxy-selenide, respectively, compared with the group that received acetylselenide. Similarly, in the absence of Se foliar supply and potassium hydroxy-selenide, Nugrain430 decreased MDA content by 39.40% and 28.98%, respectively compared with acetylselenide.

In BM737, Figure 1C shows that SOD levels increased by 13.99%, 24.34%, and 20.06% with sodium selenate compared with the absence Se foliar supply, potassium hydroxy-selenide, and acetylselenide, respectively. Moreover, K200 demonstrated lower SOD levels with potassium hydroxy-selenide, with reductions of 18.19%, 22.55%, and 21.81% without Se foliar supply, sodium selenate, and acetylselenide, respectively.

The CAT activity was highest (Figure 1D) in the Enforcer with potassium hydroxy-selenide, which showed remarkable enhancement compared with treatments without Se foliar supply, sodium selenate, and acetylselenide by 43.40%, 26.42%, and 21.70%, respectively. In the absence of Se foliar supply, sodium selenate, and potassium hydroxy-selenide, Nugrain320 demonstrated an increase in CAT activity by 44.19%, 37.66%, and 31.43%, respectively, when treated with acetylselenide. Similarly, SHS410 exhibited increased CAT activity of 39.39%, 33.33%, and 29.41% without Se foliar supply, sodium selenate, and potassium hydroxy-selenide, respectively, when treated with acetylselenide.

For most genotypes, applying selenium in the form of potassium hydroxy-selenide, sodium selenate, and acetylselenide increased CAT activity compared with plants without Se foliar supply. Nugrain420 exhibited a positive response to Se fertilization showed a more significant increase in CAT activity, with improvements of 25.00%, 23.94% and 21.74% with potassium hydroxy-selenide, sodium selenate, and acetylselenide, respectively when compared with the treatment without Se foliar supply. Among the three forms of selenium, sodium selenate and acetylselenide showed a more significant increase in CAT activity in BRS310, with an improvement of 21.87% and 21.05%, respectively. However, the Nugrain430 with sodium selenate showed a more significant increase in CAT activity, with improvements of 52.83% and 58.49% for potassium hydroxy-selenide and acetylselenide, respectively.

Regarding APX activity, sodium selenate was the most effective form of selenium in SHS410, with an improvement of 15.03%, 47.49%, and 56.94% compared with absence Se foliar supply, potassium hydroxy-selenide, and acetylselenide, respectively. In BRS310, sodium selenate, potassium hydroxy-selenide, and acetylselenide increased APX activity by 19.48%, 30.21%, and 24.04%, respectively, compared with the treatment without Se foliar supply. For Nugrain320, treatment with sodium selenate, potassium hydroxy-selenide, and acetylselenide increased APX activity by 36.46%, 29.02%, and 32.76%, respectively, compared with the treatment without Se foliar supply. Compared with potassium hydroxy-selenide, K200 exhibited a more significant decrease in APX activity, with reductions of 55.51%, 57.98%, and 43.95%, respectively, when compared with treatment without Se foliar supply, sodium selenate and acetylselenide. Lastly, Nugrain420 showed a decrease in APX activity of 18.23%and 24.60%, respectively, when the treatments potassium hydroxy-selenide and acetylselenide are compared with the treatment without Se foliar supply.

Potassium hydroxy-selenide enhanced protein content in Nugrain420 by 12.60%, 13.14%, and 18.80% compared with the treatments without Se foliar supply, sodium selenate, and acetylselenide, respectively. Similarly, Nugrain430 increased the protein content by 16.49%, 19.97%, and 36.88% when treated with potassium hydroxy-selenide compared with the treatments without Se foliar supply, sodium selenate, and acetylselenide, respectively. In BM737, treatment without Se foliar supply, with sodium selenate, or with potassium hydroxy-selenide increased protein content of 28.52%, 35.60%, and 31.08%, respectively, compared with the treatment with acetylselenide. However, when K200 was treated with sodium selenate, potassium hydroxy-selenide, and acetylselenide, its protein content decreased by 22.67%, 30.48%, and 19.76%, respectively, when compared with the treatment without Se foliar supply.

### 3.2. Selenium and Nutritional Efficiency

**Figure 2:**
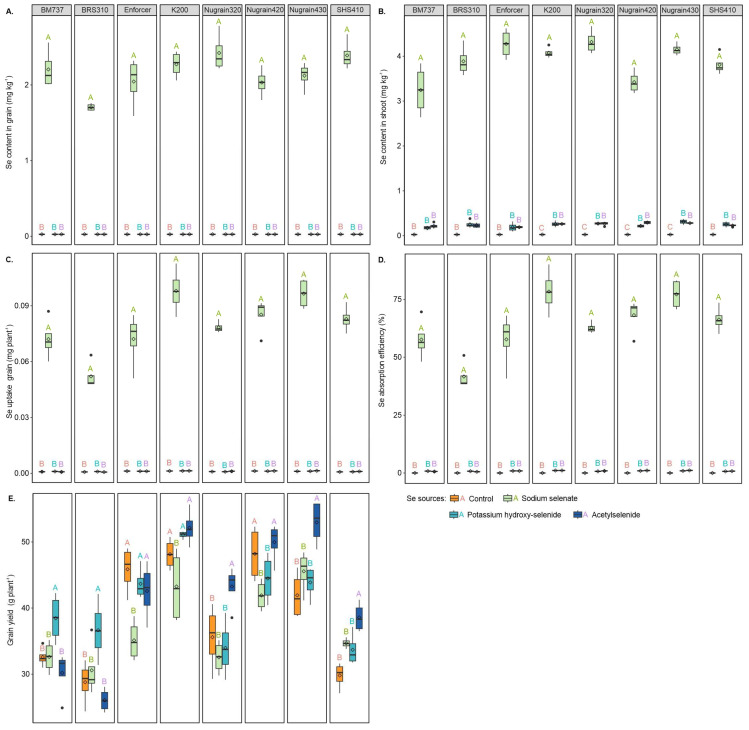
Selenium content in grain (**A**), Se content in the shoot (**B**), Se uptake by grain (**C**), Se absorption efficiency (**D**), grain yield (**E**). Se sources with the same genotype are compared using uppercase letters. Using the Scott–Knott test, different letters indicate significant differences between treatments at a probability threshold of 5% (*p* < 0.05).

The use of organoselenium compounds, including potassium hydroxy-selenide and acetylselenide, resulted in minimal levels of Se detected in the leaves and undetectable Se contents in the grain. In contrast, a foliar spray of sodium selenate led to a significant increase in Se content in both grain and shoot, as shown in Figure 2A,B. Furthermore, this treatment enhanced Se uptake by the grain and increased Se absorption efficiency, as indicated in Figure 2C,D.

### 3.3. Grain Yield

The graph (Figure 2E) shows that grain yield varied among different genotypes and that Se fertilization positively impacted yield, overall. In BM737, the yield for plants treated with potassium hydroxy-selenide increased by 15.26%, 15.21%, and 21.50% compared with the treatments without Se foliar supply, sodium selenate, and acetylselenide, respectively. Similarly, in BRS310, potassium hydroxy-selenide increased yield by 21.44%, 16.55%, and 28.90% compared with the same previously mentioned treatments, respectively. For Enforcer, the treatment without Se foliar supply potassium hydroxy-selenide, and acetylselenide resulted in higher yields than, sodium selenate, respectively, with increases of 23.42%, 23.42%, and 17.51%. Furthermore, K200 showed yield increases of 10.17%, 15.30%, and 17.07% for the treatments without Se foliar supply, potassium hydroxy-selenide, and acetylselenide, when compared with the treatment using sodium selenate.

In general, adding acetylselenide showed significant improvements in grain yield compared with other selenium treatments. When acetylselenide was used in Nugrain320, there were yield increases of 17.69%, 24.75%, and 22.82% compared with absence Se foliar supply, sodium selenate, potassium hydroxy-selenide, respectively. Nugrain430 plants without Se foliar supply or treated with sodium selenate and potassium hydroxy-selenide exhibited lower grain yields of 20.83%, 13.98%, and 17.16% compared with acetylselenide. When comparing SHS410 with acetylselenide to treatments without Se foliar supply, sodium selenate, and potassium hydroxy-selenide, there were yield increases of 22.76%, 10.14%, and 12.70%, respectively. Lastly, compared with sodium selenate and potassium hydroxy-selenide, Nugrain420 showed increases of 16.13% and 11.00%, respectively, with acetylselenide but did not significantly differ from the treatment without Se foliar supply.

### 3.4. Macronutrient Content

**Figure 3:**
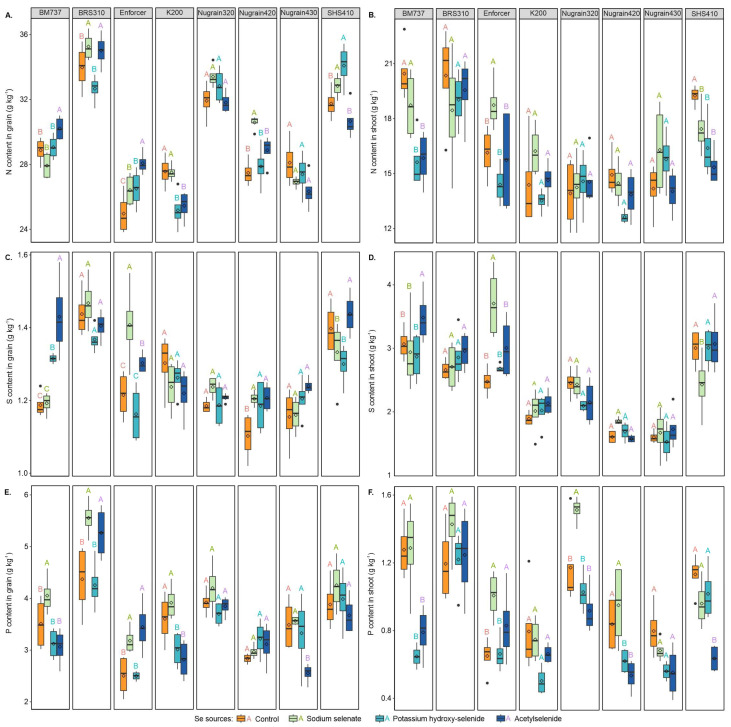
N content in grain (**A**), N content in shoot (**B**), S content in grain (**C**), S content in shoot (**D**), P content in grain (**E**) and P content in shoot (**F**) of the sorghum plants. Se sources with the same genotype are compared using uppercase letters. Using the Scott-Knott test, different letters indicate significant differences between treatments at a probability threshold of 5% (*p* < 0.05).

Significant differences in the nitrogen content in grain (Figure 3A) were observed in BM737, BRS310, Enforcer, K200, SHS410, and Nugrain420 across different selenium sources and genotypes. Nugrain320 and Nugrain430 did not exhibit significant responses to various selenium sources. The N content in the shoot (Figure 3B) with Enforcer, compared with treatments without Se foliar supply, potassium hydroxy-selenide, and acetylselenide, the decreased by 13.85%, 23.16%, and 16.05%, respectively, when treated with sodium selenate and in BM737, potassium hydroxy-selenide, and acetylselenide increased N content in the shoot by 16.59% and 15.42%, respectively, compared with sodium selenate.

For SHS410, the reduction in N content in the shoot for treatments sodium selenate, potassium hydroxy-selenide, and acetylselenide was 9.43%, 14.86%, and 20.29%, respectively, when compared with the control, i.e., without Se foliar supply. The selenium treatments did not significantly affect the N content in the shoots of BRS310, K200, Nugrain320, Nugrain420, and Nugrain430.

BM737 treated with acetylselenide showed a significant increase in S content in the grain (Figure 3C) by 16.96%, 16.60%, and 8.04%, respectively, compared with treatments without Se foliar supply, sodium selenate, and potassium hydroxy-selenide. Comparing SHS4100 treated with acetylselenide to sodium selenate and potassium hydroxy-selenide, S contents in the grain increased by 7.30% and 9.56%, respectively. In Enforcer, compared with treatments without Se foliar supply, potassium hydroxy-selenide, and acetylselenide, there were increases in S content in the grain of 13.67%, 17.41%, and 7.46%, respectively, when treated with sodium selenate. Nugrain420 showed increased S content in the grain by 8.51%, 6.96%, and 8.69% when treated with sodium selenate, potassium hydroxy-selenide, and acetylselenide compared with treatments without Se foliar supply. BRS310, K200, Nugrain320, and Nugrain430 did not show any significant Se effect on the S content of the grain.

When treated with acetylselenide, the S content in the shoot (Figure 3D) of genotype BM737 increased significantly by 12.05%, 15.71%, and 17.72%, respectively, compared with treatments without Se foliar supply, sodium selenate, and potassium hydroxy-selenide. In Enforcer, compared with treatments without Se foliar supply, potassium hydroxy-selenide, and acetylselenide, there were increases in S content in the shoot of 33.13%, 27.66%, and 18.89%, respectively, when plants were treated with sodium selenate. Compared with the treatment sodium selenate, SHS410 showed increases of 19.07%, 19.20%, and 20.78% for treatments without Se foliar supply, potassium hydroxy-selenide, and acetylselenide, respectively. Selenium sources did not significantly influence the S content in the shoot in BRS310, K200, Nugrain320, Nugrain420, or Nugrain430.

The content of P in grain (shown in Figure 3E) increased significantly with the addition of sodium selenate to BM737, with increases of 13.45%, 22.99%, and 24.41% observed in the absence of Se foliar supply, potassium hydroxy-selenide, and acetylselenide treatments, respectively. Compared with the treatment without Se foliar supply, the BRS310 genotype showed increases of 21.28% and 16.99% with sodium selenate and acetylselenide, respectively. Similarly, compared with the treatment without Se foliar supply, the Enforcer genotype showed increases of 21.37% and 27.41% with sodium selenate and acetylselenide, respectively. The Nugrain430 genotype exhibited increases in the P content in the grain of 26.93%, 28.57%, and 23.42% with the treatments without Se foliar supply, sodium selenate, and potassium hydroxy-selenide treatments, respectively, compared with acetylselenide,. Finally, compared with the absence of Se foliar supply, K200 showed a decrease in the P content in the grain of 16.44% and 21.91% with potassium hydroxy-selenide and acetylselenide treatments, respectively.

When potassium hydroxy-selenide and acetylselenide were applied to BM737 and Nugrain420, there was a reduction in P content in the shoot (shown in Figure 3F). In contrast, Nugrain320 treated with sodium selenate treatment showed an increase in P content in the shoot of 22.48%, 32.06%, and 39.34% compared with the treatment without Se foliar supply, potassium hydroxy-selenide, and acetylselenide, respectively. However, when acetylselenide was used, the shoot’s P content decreased by 43.93%, 33.85%, and 37.59% compared with the treatment without Se foliar supply, sodium selenate, and potassium hydroxy-selenide, respectively, in the case of SHS410.

**Figure 4:**
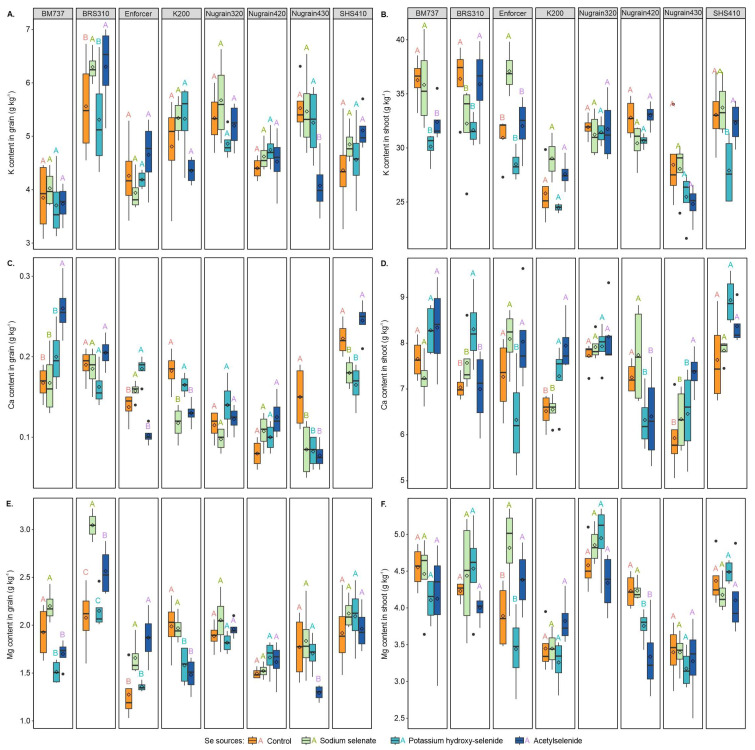
K content in grain (**A**), K content in shoot (**B**), Ca content in grain (**C**), Ca content in shoot (**D**), Mg content in grain (**E**) and Mg content in shoot (**F**) of the sorghum plants. Se sources with the same genotype are compared using uppercase letters. Using the Scott-Knott test, different letters indicate significant differences between treatments at a probability threshold of 5% (*p* < 0.05).

Adding Se foliar resulted in significant increases in K content in both grain and shoot of sorghum. More specifically, the K content in the grain of genotype BRS310 increased by 11.67% and 11.78% with sodium selenate and acetylselenide, when compared with absence of Se foliar supply (Figure 4A). Acetylselenide led to remarkable decreases in K content in the grain of Nugrain430, with reductions of 26.24%, 25.43%, and 22.42% being observed when contrasted with treatments without Se foliar supply, sodium selenate, and potassium hydroxy-selenide, respectively.

For sorghum shoots, potassium hydroxy-selenide, and acetylselenide increased K content (Figure 4B) by 16.93% and 10.66% in BM737 and by 11.37% and 13.03% in BRS310 with sodium selenate and potassium hydroxy-selenide treatments when contrasted with the treatment without Se foliar supply. In Enforcer, adding sodium selenate increased K content in the shoot by 16.37%, 23.16%, and 13.65%, respectively, when compared with treatments without Se foliar supply, potassium hydroxy-selenide, and acetylselenide. Lastly, SHS410 showed a significant decrease in K content in the shoot, with potassium hydroxy-selenide, leading to reductions of 15.56%, 17.25%, and 13.59% when compared with the absence of Se foliar supply, or the addition of sodium selenate or acetylselenide, correspondingly.

In BM737, the addition of acetylselenide significantly increased Ca content in grain (Figure 4C) by 35.58%, 35.58%, and 23.08%, when compared with treatments without Se foliar supply, sodium selenate, and potassium hydroxy-selenide, respectively. SHS410 also showed a substantial increase in Ca content in the grain, with acetylselenide leading to increases of 26.53% and 32.65% compared with sodium selenate and potassium hydroxy-selenide. Moreover, K200 exhibited an increase of 28.78% and 21.21% in Ca content in the grain with the addition of potassium hydroxy-selenide when contrasted with sodium selenate and acetylselenide. Finally, for Nugrain430, a greater Ca content in the grain was observed with the treatment without Se foliar supply, which showed values 43.33%, 45.00%, and 48.33% higher when compared with sodium selenate, potassium hydroxy-selenide, and acetylselenide, respectively.

In BRS310, adding potassium hydroxy-selenide led to a significant increase in Ca content in the shoot (Figure 4D)—by 15.29%, 8.85%, and 15.74%—compared with treatments without Se foliar supply, sodium selenate and acetylselenide, respectively. Nugrain430 exhibited significant increases in Ca content in the shoot with the acetylselenide treatment, showing increases of 19.57%, 13.97%, and 12.38% compared with treatments without Se foliar supply, sodium selenate and potassium hydroxy-selenide, respectively. In K200, Ca content in the shoot increased by 10.51% and 18.02% with potassium hydroxy-selenide and acetylselenide treatments, respectively, compared with the treatment without Se foliar supply. Regarding the potassium hydroxy-selenide treatment, the Enforcer genotype showed decreases of 12.94%, 21.84%, and 21.26% compared with the treatment without Se foliar supply, sodium selenate, and acetylselenide, in that order. Finally, Nugrain420 improved Ca content in the shoot by 12.82% for potassium hydroxy-selenide and by 11.72% for acetylselenide treatments in comparison with the treatment without Se foliar supply.

The Mg content in the grain (Figure 4E) showed greater values—i.e., 31.25% and 22.72%—for treatments without Se foliar supply and sodium selenate in BM737, and 19.69% and 24.65% in K200, respectively, compared with potassium hydroxy-selenide and acetylselenide. Sodium selenate in BRS310 increased Mg content in the grain by 31.77%, 29.39%, and 15.76% compared with treatments without Se foliar supply, potassium hydroxy-selenide, and acetylselenide, respectively. The genotype Enforcer showed an increase in Mg content in the grain of 23.07% and 18.09% with sodium selenate and 31.81% and 27.41% with acetylselenide, respectively compared with the treatment without Se foliar supply and potassium hydroxy-selenide. Nugrain430 revealed increases of 26.97%, 29.56%, and 24.19% for treatments without Se foliar supply, sodium selenate, and potassium hydroxy-selenide, respectively, when contrasted with acetylselenide. Lastly, in the shoots, sodium selenate in Nugrain420 increased Mg content (Figure 4F) by 11.33% and 21.19% compared with potassium hydroxy-selenide and acetylselenide, respectively, whereas Enforcer showed an increase of 19.25% and 28.59% with sodium selenate, and 11.24% and 21.54% with acetylselenide compared with treatments without Se foliar supply and potassium hydroxy-selenide, in that order.

### 3.5. Micronutrient Content

**Figure 5:**
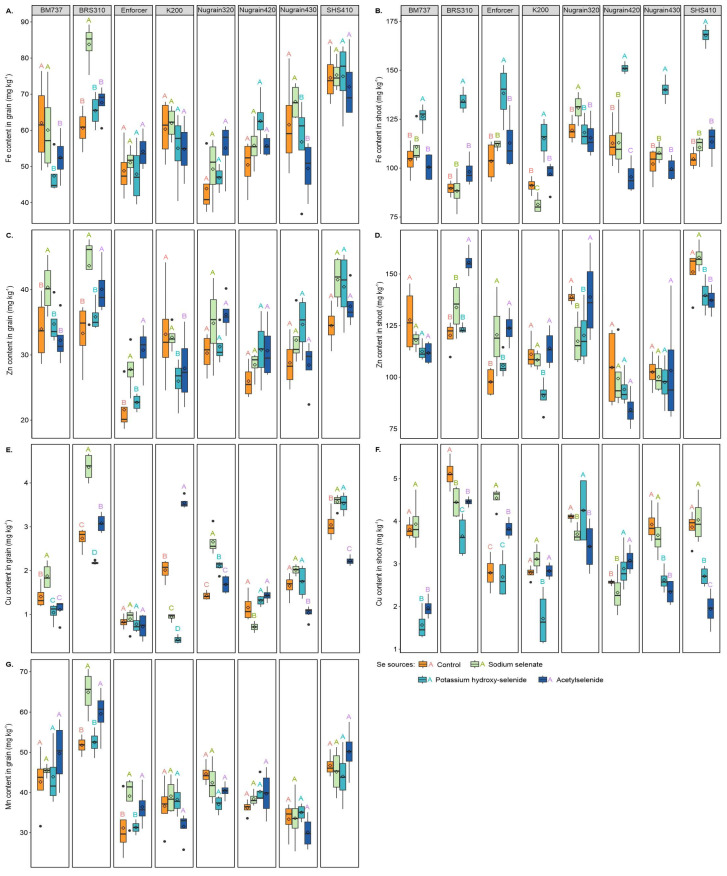
Fe content in grain (**A**), Fe content in shoot (**B**), Zn content in grain (**C**), Zn content in shoot (**D**), Cu content in grain (**E**), Cu content in shoot (**F**) and Mn content in grain (**G**) of the sorghum plants.Se sources with the same genotype are compared using uppercase letters. Using the Scott-Knott test, different letters indicate significant differences between treatments at a probability threshold of 5% (*p* < 0.05).

The application of sodium selenate in BRS310 resulted in a significant increase in the Fe content in the grain (Figure 5A), with increments of 27.64%, 21.89%, and 19.2% being observed for this treatment when compared with the treatments without Se foliar supply, potassium hydroxy-selenide, and acetylselenide, respectively. In BM737, sodium selenate increased Fe content in the grain by 21.11% and 12.63% compared with potassium hydroxy-selenide and acetylselenide, in that order. In Nugrain430, sodium selenate increased Fe content in the grain by 16.39% and 27.20% compared with potassium hydroxy-selenide and acetylselenide, respectively.

Among the treatments, potassium hydroxy-selenide demonstrated the highest increase in Fe content in the shoots (Figure 5B) of SHS410, with a 37.61%, 34.08%, and 32.39% increment compared with the treatments without Se foliar supply, sodium selenate, and acetylselenide, respectively. In Nugrain320, sodium selenate increased the Fe content in the shoot by 8.74%, 9.60%, and 11.67%, respectively, compared with the treatments without Se foliar supply, potassium hydroxy-selenide, and acetylselenide.

As shown in Figure 5C, treating BRS310 with sodium selenate resulted in a higher Zn content in the grain compared with the treatments without Se foliar supply and potassium hydroxy-selenide. More specifically, the increase in Zn content in the grain was 23.73% and 17.92%, respectively. Similar trends were observed for genotype Enforcer, for which the treatment with sodium selenate led to increases of 22.30% and 18.31% in Zn content in the grains when compared with the treatments without Se foliar supply and with potassium hydroxy-selenide. Treating K200 with sodium selenate also led to increases of 20.55% and 14.57% in grain Zn content when compared with potassium hydroxy-selenide and acetylselenide. In BM737, the application of sodium selenate resulted in Zn contents in the grain that were 15.84%, 13.92%, and 20.13% higher than those obtained with potassium hydroxy-selenide, acetylselenide, and without Se foliar supply, respectively.

In shoots, sodium selenate showed a greater effect on Zn content (Figure 5D) in SHS410 plants, which were 11.65% and 13.17% higher than for potassium hydroxy-selenide and acetylselenide, respectively. In Nugrain320, acetylselenide showed a significant increase in Zn content in the shoot compared with sodium selenate and potassium hydroxy-selenide. BRS310 treated with acetylselenide showed increases in Zn content in the grains by 22.75%, 14.06%, and 20.77% compared with no Se foliar supply, sodium selenate, and potassium hydroxy-selenide, in that order. Acetylselenide applied to Enforcer increased Zn content in the grain by 21.02% and 14.52%, while sodium selenate resulted in increases of 18.89% and 12.22%, respectively, when these treatments were contrasted with no Se foliar supply and potassium hydroxy-selenide. Genotype K200 showed an increase in Zn content by 18.10%, 16.02%, and 20.19% with no Se foliar supply, sodium selenate, and acetylselenide, respectively, when compared with potassium hydroxy-selenide.

Regarding Cu in the grain (Figure 5E), sodium selenate increased Cu content in BM737 by 24.93%, 44.40%, and 40.93% compared with no Se foliar supply, potassium hydroxy-selenide, and acetylselenide, respectively. In BRS310, sodium selenate increased Cu content in the grain by 37.24%, 49.97%, and 29.26% compared with no Se foliar supply, potassium hydroxy-selenide, and acetylselenide, correspondingly. In Nugrain320, sodium selenate increased Cu content in the grain by 46.59%, 21.35%, and 36.59% compared with no Se foliar supply, potassium hydroxy-selenide, and acetylselenide, in that order.

In the case of Cu in grains of genotype SHS410, when compared with the absence of Se foliar supply and with acetylselenide, sodium selenate led to increases of 14.77% and 37.75%, while potassium hydroxy-selenide resulted in 13.87% and 37.08% more Cu in the grains, respectively. Similarly, Nugrain420’s application of sodium selenate resulted in 38.23%, 45.52%, and 50.35% greater Cu contents in the grain compared with the treatments without Se foliar, potassium hydroxy-selenide, and acetylselenide, respectively. For Nugrain430, the increase of 37.42%, 48.76%, and 40.91% was observed with the application of acetylselenide compared with the treatments without Se foliar supply, sodium selenate, and potassium hydroxy-selenide, in that order. Furthermore, K200 showed significant increases of 43.38%, 73.39%, and 87.96% with the application of acetylselenide compared with the treatment without Se foliar supply, sodium selenate, and potassium hydroxy-selenide.

Concerning Cu in the shoots (Figure 5F), sodium selenate resulted in increments of 28.59% and 36.35% in Nugrain430, 32.75% and 51.86% in SHS410, and 60.18% and 49.90% in BM737, when compared with potassium hydroxy-selenide and acetylselenide, respectively. Additionally, the application of sodium selenate in Enforcer resulted in increments of 38.52%, 40.73%, and 15.85% when compared with the treatment without Se foliar supply, potassium hydroxy-selenide, and acetylselenide, correspondingly. Furthermore, in Nugrain320, the application of potassium hydroxy-selenide resulted in 20.15% more Cu in the shoots compared with sodium selenate and a 19.85% increase compared with acetylselenide. With the addition of potassium hydroxy-selenide, Nugrain420 saw an improvement of 11.16% and 19.46% in shoot Cu, while acetylselenide increased it by 16.64% and 24.43% when compared with the control (without Se foliar supply) and sodium selenate, respectively. Among the treatments, K200 saw the most significant decrease in shoot Cu content following the application of potassium hydroxy-selenide, with reductions of 38.38%, 44.86%, and 39.36% compared with the untreated Se foliar supply, sodium selenate, and acetylselenide, respectively. BRS310 with absence of Se foliar supply had Cu shoot contents 13.09%, 28.63%, and 12.95% greater when compared with sodium selenate, potassium hydroxy-selenide, and acetylselenide respectively,

Finally, adding sodium selenate increased the Mn content in grain (Figure 5G) in BRS310 by 20.25% and 19.15%, while acetylselenide increased it by 13.11% and 11.91% when compared with the untreated Se foliar supply and with potassium hydroxy-selenide. Similarly, the genotype Enforcer exhibited increments in Mn content in the grain of 20.32% and 19.92% with sodium selenate and 14.40% and 13.98% with acetylselenide, respectively, when compared with the untreated Se foliar supply and with potassium hydroxy-selenide.

### 3.6. Principal Component Analysis

**Figure 6:**
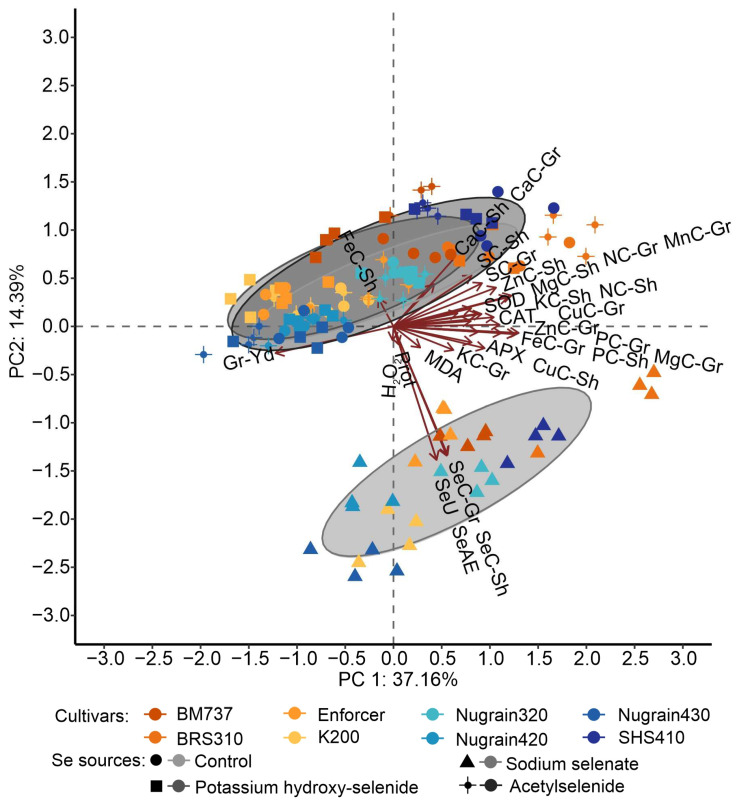
Principal component analysis. Abbreviations: Se content in grain (SeC-Gr) and in the shoot (SeC-Sh); Se absorption efficiency (SeAE); Se uptake (SeU); lipid peroxidation (MDA); hydrogen peroxide (H_2_O_2_); catalase (CAT); ascorbate peroxidase (APX), superoxide dismutase (SOD), proteins in extract enzymatic (Prot); grain yield (Gr-Yd); N (NC-Gr), S (SC-Gr), P (PC-Gr), K (KC-Gr), Mg (MgC-Gr), Fe (FeC-Gr), Zn (ZnC-Gr), Mn (MnC-Gr), Cu (CuC-Gr) content in the grain; N (NC-Sh), S (SC-Sh), P (PC-Sh), K (KC-Sh), Mg (MgC-Sh), Ca (CaC-Sh); Fe (FeC-Sh); Zn (ZnC-Sh), Mn (MnC-Sh) and Cu (CuC-Sh) content in the shoot.

Figure 6 shows the results of the principal component analysis, which allowed us to gain a deeper understanding of how different sources of selenium and genotypes influenced the variables under investigation. The first component, which represented most of the overall change, was plotted along the horizontal axis. Based on these results, we have created clusters based on selenium sources and genotypes. The first two principal components explained 51.55% of the overall variance. Our findings indicated that potassium hydroxy-selenide and acetylselenide sources significantly impacted grain yield, particularly for Nugrain430 and SHS410. On the other hand, applying Se as sodium selenate led to a more substantial response regarding Se content relative to genotypes.

## 4. Discussion

Plants have developed various strategies to combat oxidative stress, such as producing reactive oxygen species (ROS) in response to environmental conditions [41]. To maintain ROS levels within a safe range, cells employ enzymatic and non-enzymatic antioxidant mechanisms [41]. However, the results of sorghum genotypes treated with selenium were inconsistent, with increased and decreased H_2_O_2_ and MDA levels observed. This could be attributed to the fact that a phytotoxic content of selenium may have been reached, even though visible signs of phytotoxicity were absent. According to Ríos et al. [42], when selenium concentrations reach phytotoxic levels, it increases the H_2_O_2_ content, leading to an elevation in MDA levels.

Selenium has been shown to enhance the antioxidant capacity of plant cells by increasing the activity of antioxidant enzymes, thereby improving plant resistance to stress [43]. According to Shieber and Chandel [44], superoxide dismutase (SOD) is the primary antioxidant enzyme that operates as a barrier against oxidative stress by catalyzing the O_2_-dismutation process, which generates O_2_ and H_2_O_2_. Meanwhile, catalase (CAT) breaks down H_2_O_2_ to H_2_O and O_2_, thanks to its high affinity for this substrate. Additionally, ascorbate peroxidase (APX) utilizes ascorbate as an electron donor to convert H_2_O_2_ into H_2_O and O_2_ [11,45].

The organoselenium compound acetylselenide showed low selenium contents in the plants, yet it increased the antioxidant action. Selenium-containing compounds were designed and synthesized based on their ability to scavenge oxidants [21]. An example of the best-known synthetic compound that can counteract oxidative species is Ebselen, which has high antioxidants [46]. Similar activities were exhibited by some water-soluble selenides [47]. Organoselenium compounds counteract lipid peroxidation and free radical chain reaction [48].

Low levels of selenium (Se) have been found to protect against various abiotic stressors, including drought, cold, heat, salinity, and UV-B radiation, all of which cause oxidative stress [43]. A study by Djanaguiraman et al. [49] showed that Se applied via foliar spraying had a beneficial effect on sorghum plants under high-temperature stress by reducing oxidative stress and membrane damage through the intensification of antioxidant defense mechanisms in sorghum grain. However, soils with low Se content can result in food with insufficient Se content, which can adversely affect the health of animals and humans [50]. Therefore, the biofortification of Se is essential, and its efficacy has been demonstrated by several authors [12,51,52,53]. In this study, foliar fertilization using selenate proved to be the most effective method for Se biofortification in sorghum compared with the other sources tested.

Additional sources of selenium, such as SeSO_4_, have also been investigated for their efficacy in promoting sorghum plant growth, with positive results reported [54]. Kikkert and Berkelaar [55] assessed the mobility of selenium in canola and wheat by measuring the translocation factor. They found that selenate exhibited the highest mobility, followed by SeMet, and selenite/SeCys. Leaves can absorb selenium through active transport via the symplastic pathway, after which it is transported long distances to other tissues through the phloem vascular system [56,57]. However, the absorption, translocation, and distribution of selenium are influenced by various factors such as plant species, growth stage, form and concentration of Se, presence of other substances, the activity of membrane transporters, and plant translocation mechanisms [58,59,60].

The use of selenate as a source of selenium has been shown to increase the selenium content in the grain of all genotypes studied. However, it is essential to consider the maximum permissible limit of selenium intake. For instance, the recommended daily selenium intake for adults ranges from 30 to 70 µg day^−1^ [61], while for animals such as dairy cattle, the recommended intake is 0.3 mg kg^−1^ dry mass (DM) based diet [62]. For beef cattle, broilers, and swine, the recommended selenium intake ranges from 0.1 to 0.3, 0.1 to 0.15, and 0.1 to 0.30 mg kg^−1^ of diet, respectively [63,64,65]. Thus, careful attention must be paid to the amounts of selenium added to their diet to avoid exceeding the recommended limits for human and animal consumption.

The application of low doses of selenium has been shown to increase yield, possibly due to its positive effects on plant growth and development [66]. By inhibiting senescence and enhancing the antioxidant defense system, selenium modifies the activity of enzymes such as catalase (CAT) and superoxide dismutase (SOD), which may account for the observed productivity gains [67]. For instance, when Ducsay et al. [36] administered 10 g ha^−1^ of selenate through the leaf, they observed a significant increase in wheat yield compared with the control group. These findings suggest that carefully controlled selenium supplementation can be a useful tool for increasing agricultural productivity.

Selenium (Se) and sulfur (S) exhibit chemical similarities, and therefore, the primary mode of Se absorption and assimilation is with S transporters and enzymes. This process can influence the N assimilation pathway. Furthermore, Se can affect the metabolic pathways of S, N, and phenol, with the degree of impact depending on the rate of Se, the type of Se (selenate/sulfate), and the duration of exposure to Se fertilization. These factors must be considered when studying Se’s effects on these pathways [12].

The utilization of Se may hinder the absorption of certain nutrients, including P, K, Mg, and Fe, due to their abundance in sorghum [68]. However, the positive effects of Se on Fe absorption have been well-documented [69,70]. This mechanism of Se-mediated reduction of metal toxicity in plants is noteworthy [71]. Zinc is an essential component of the CuZnSOD enzyme, which is vital in protecting against ROS attacks. It also acts as an antagonist with copper and iron, preventing the oxidation of sulfhydryl groups in proteins [72].

## 5. Conclusions

Biofortification with selenium increased the yield of sorghum plants. Supplementation with selenium through sodium selenate was more efficient than organoselenium compounds. Acetylselenide had a positive effect on the antioxidant system. Sorghum can be biofortified through foliar application with sodium selenate. Studying the interaction between organic and inorganic compounds in plants is necessary.

## Data Availability

Data is contained within the article or Supplementary Material.

## Supplementary Materials

The following supporting information can be downloaded at: https://www.mdpi.com/article/10.3390/foods12102034/s1, Table S1: Summary of characteristics of eight sorghum cultivars; Table S2: Chemical characterization and particle size distribution before sowing the soil used in the greenhouse-grown.; Table S3: Lines used to determine the elements with ICP-OES and assessment of precision through the analysis of Peach Leaves SRM1547; Table S4: Analysis of variance of eight sorghum genotypes cultivated in greenhouse conditions, fertilized with different Se sources. Reference [73] is cited in Supplementary Materials.
