# Supplementary material for: Sodium Selenate, Potassium Hydroxy-Selenide, Acetylselenide and Their Effect on Antioxidant Metabolism and Plant Nutrition and Yield in Sorghum Genotypes
Source: Foods. 2023 May 17;12(10):2034. doi: 10.3390/foods12102034 (PMC10217391; doi:10.3390/foods12102034)
Supplement: Supplementary file 1 [file foods-12-02034-s001.zip › foods-2315300-supplementary.pdf]

Supplementary Material

Table S1. Summary of characteristics of eight sorghum cultivars.

Cultivars	RNC*	PH (cm)	SF (days)	SH (days)	Color grain
BM737	29344	135-145	51-55	120-130	Red
BRS310	18751	115	65	120	Red
Enforcer	30336	120-130	55-60	110-120	Brown
K200	38142	140-160	UN	UN	Red
Nugrain320	38145	130	58-60	110-115	Orange
Nugrain420	40315	150	65-70	125-130	Orange
Nugrain430	36743	140	65-70	125-130	Orange
SHS410	18772	125-135	48-52	120-130	Red

UN – uninformed; PH – plant height; SF – sowing to flowering; SC – sowing to harvest. *RNC- National Registry of cultivars. Details about patent and Maintainer of cultivar can be found at Brazil [73].

Table S2. Chemical characterization and particle size distribution before sowing of the soil used in the greenhouse-grown.

	Units	Method/ Extractant	Greenhouse
Sand	g kg^{-1}	Hydrometer	730
Silt	g kg^{-1}	Hydrometer	50
Clay	g kg^{-1}	Hydrometer	220
SOM	g kg^{-1}	$Na_2Cr_2O_7$ 4N + H_2SO_4 10N	6.8
pH		Water 1:2.5	5.1
Al	$cmol_c$ dm^{-3}	KCl (1 mol L^{-1})	0.45
Al+H	$cmol_c$ dm^{-3}	SMP	2.37
P	mg dm^{-3}	Mehlich-1	0.71
K	mg dm^{-3}	Mehlich-1	23.46
Ca	$cmol_c$ dm^{-3}	KCl (1 mol L^{-1})	0.19
Mg	$cmol_c$ dm^{-3}	KCl (1 mol L^{-1})	0.10
S	mg dm^{-3}	$Ca(H_2PO_4)_2$ + CH_3COOH	4.23
B	mg dm^{-3}	Hot water	0.07
Cu	mg dm^{-3}	Mehlich-1	0.10
Fe	mg dm^{-3}	Mehlich-1	47.33
Mn	mg dm^{-3}	Mehlich-1	2.44
Zn	mg dm^{-3}	Mehlich-1	0.28
Se	mg kg^{-1}	USEPA3051A	<DL

SOM – soil organic matter; DL – detection limit.

Table S3. Lines used to determine the elements with ICP-OES and assessment of precision through the analysis of Peach Leaves SRM1547.

Element or Nutrient	λ (nm)	Certified[a] (mg kg^{-1})	Found[b] (mg kg^{-1})	% Recovery[c]
S	180.731	2000	1566±501	78
P	178.287	1371±82	1343±43	98
K	769.896	24330±380	25584±528	105
Ca	315.887	15590±160	16948±501	109
Mg	279.079	4320±150	4586±139	106
Fe	373.486	219.8±6.8	214±10,9	97
Cu	324.754	3.75±0.37	2.70±0.22	72
Zn	213.856	17.97±0.53	17.3±0.96	96
Mn	403.076	97.8±1.8	113±2.39	116

[a] Results for Peach Leaves SRM1547 represented as mean ± confidence interval, informative value. [b] Mean ± standard error of the mean. Average of ten determinations. [c] Average of ten determinations.

Table S4. Analysis of variance of eight sorghum cultivars cultivated in greenhouse and in field conditions, fertilized with different Se sources.

Source of variation	F-test			
	C	S	CxS	CV
H_2O_2	*	ns	**	16.63
MDA	**	**	**	13.14
SOD	**	*	*	12.21
CAT	**	**	**	16.69
APX	**	**	**	15.53
Protein	**	*	**	14.90
SeC-Gr	**	**	**	19.57
SeC-Sh	**	**	**	14.33
SeU	**	**	**	23.46
SeAE	**	**	**	23.75
Grain yield	**	**	**	8.21
Grain Weight	**	ns	ns	15.69
NC-Gr	**	*	**	3.45
SC-Gr	**	**	**	4.91
PC-Gr	**	**	**	11.57
KC-Gr	**	ns	*	12.85
CaC-Gr	**	**	**	16.45
MgC-Gr	**	**	**	12.53
FeC-Gr	**	**	*	13.52
ZnC-Gr	**	**	*	13.02
CuC-Gr	**	**	**	12.47
MnC-Gr	**	*	*	12.10
NC-Sh	**	**	*	10.85
SC-Sh	**	ns	**	13.60
PC-Sh	**	**	**	18.55
KC-Sh	**	**	**	8.12
CaC-Sh	**	*	**	9.79
MgC-Sh	**	*	*	10.77
FeC-Sh	**	**	**	7.52
ZnC-Sh	**	**	**	9.88
CuC-Sh	**	**	**	12.38
MnC-Sh	**	ns	ns	11.31

ns – not significant by F-test; * - significant by F-test at $p<0,05$; ** - significant by F-test at $p<0,01$;
CV – coefficient of variation (%).
Degrees of freedom, greenhouse: cultivars (C) – 7; doses (D) – 3; C x D – 21; residue – 96
Abbreviations: Se content in grain (SeC-Gr) and in shoot (SeC-Sh); Se absorption efficiency (SeAE); Se uptake (SeU); lipid peroxidation (MDA); hydrogen peroxide (H_2O_2); catalase (CAT); ascorbate peroxidase (APX), superoxide dismutase (SOD); N (NC-Gr), S (SC-Gr), P (PC-Gr), K (KC-Gr), Mg (MgC-Gr), Fe (FeC-Gr), Zn (ZnC-Gr), Mn (MnC-Gr), Cu (CuC-Gr) content in the grain; N (NC-Sh), S (SC-Sh), P (PC-Sh), K (KC-Sh), Mg (MgC-Sh), Ca (CaC-Sh); Fe (FeC-Sh); Zn (ZnC-Sh), Mn (MnC-Sh) and Cu (CuC-Sh) content in the shoot.
